# Tissue-Specific Multi-Omics Integration Demonstrates Molecular Signatures Connecting Obesity to Immune Vulnerability

**DOI:** 10.3390/metabo16020095

**Published:** 2026-01-27

**Authors:** Ozge Onluturk Aydogan, Aytac Dursun Oksuzoglu, Beste Turanli

**Affiliations:** Department of Bioengineering, Faculty of Engineering, Marmara University, Istanbul 34854, Turkey; ozge.onluturk@marun.edu.tr (O.O.A.); aytac.oksuzoglu@marun.edu.tr (A.D.O.)

**Keywords:** obesity, host–pathogen interactions, systems biology, omics signatures, metabolites

## Abstract

Background: Adipose tissue surrounds organs and tissues in the body and can alter their function. It could secrete diverse biological molecules, including lipids, cytokines, hormones, and metabolites. In light of all this information, obesity can influence many tissues and organs in the body, and this situation makes obesity a central contributor to multiple disorders. It is very important to investigate the crosstalk between tissues and organs in the body to clarify the key mechanisms of obesity. Methods: In this study, we analyzed the gene expression profiles of the liver, skeletal muscle, blood, visceral, and subcutaneous adipose tissue. Differentially expressed genes (DEGs) were identified for each tissue, and functional enrichment and protein–protein interaction network analyses were performed on genes commonly identified across tissues. Priority candidate genes were identified using network-based centrality measures, and potential molecular intersection points were explored through host-pathogen interaction network analysis. This study provides an integrative framework for characterizing inter-tissue molecular patterns associated with obesity at the network level. Results: The muscle, subcutaneous adipose tissue, and blood have the highest number of DEGs. The subcutaneous adipose tissue and blood stand out due to the number of DEGs they possess, although liver and visceral adipose tissue have lower amounts. Cancer ranks first in terms of diseases associated with obesity, and this association is accompanied by leukemia, lymphoma, and gastric cancer. RPL15 and RBM39 are the top genes in both degree and betweenness metrics. The host–pathogen interaction network consists of 13 unique-host proteins, 54 unique-pathogen proteins, and 27 unique-pathogen organisms, and the Influenza A virus had the highest interaction. There were a small number of common metabolites in all tissues: 2-Oxoglutarate, Adenosine, Succinate, and D-mannose. Conclusions: In this study, we aimed to identify candidate molecules for obesity using an integrative approach, examining the gene profiles of different organs and tissues. The findings of this study suggest a possible link between obesity and immune-related biological processes. The network obtained from the host-pathogen interaction analysis, and especially the pathways associated with viral infections that stand out in the functional enrichment analysis, may overlap with molecular signatures linked to obesity. Furthermore, the co-occurrence of cytokine signaling, insulin, and glucose metabolism pathways in the enrichment results indicates that the response of cells to insulin may be affected in obese individuals, suggesting a potential interaction between immune and metabolic processes; however, further experimental validation is needed to reveal the direct functional effects of these relationships.

## 1. Introduction

Obesity is a complex metabolic disease that is defined as a body mass index (BMI) greater than 30 or an excessive amount of adipose tissue in the body. While initially a problem seen only in high-income countries, obesity is now seen as a leading disease for all regions of the world, as well as an important risk factor that paves the way for various diseases [1]. Although the World Health Organization commonly uses BMI ≥ 30 kg/m^2^ for the definition of obesity, studies report that it should be considered that these threshold values may vary among different populations and ethnic groups. In particular, some studies emphasize that this value should be lower for Asians [2,3,4]. According to a 2024 report published by the World Health Organization (WHO), there are 890 million obese adults in the world. Projections based on data from different countries have reported similar dramatic results despite using different estimation methods. If obesity can be kept at 2010 levels, the combined savings in medical expenditures will amount to a total savings of $549.5 billion over the next 20 years [5]. The obesity epidemic is predicted to peak at 31% in 2037, with a prevalence of age-standardized mean obesity in the 20–84-year-old group, on average, in the 18 European countries studied [6].

Another important concern is that many chronic complex diseases could arise due to obesity, including type 2 diabetes, cancer, and cardiovascular and liver diseases. In the instance of obesity, altered gene expression and hormonal secretion could affect the tissue and organ functions in the body. The increased adipose tissue accumulates around the tissue and organs and surrounds them over time. Therefore, it can directly communicate with and alter the tissue and organ activities [7,8]. Adipose tissue can secrete many endocrine molecules, from cytokines and lipids to microRNAs [9,10,11]. Increasing levels of Interleukin-6 (IL-6) and C-reactive protein (CRP) with obesity can lead to interrupted liver functions and may cause non-alcoholic fatty liver disease (NAFLD) or liver cancer [12,13]. It was reported that there is an association between obesity and BMI with alanine aminotransferase (ALT) and γ-glutamyl transferase (GGT) levels that are utilized for assessment of liver functions [14,15]. Leptin is another cytokine that is increased during obesity, and this increment leads to leptin resistance. Impaired leptin mechanisms may contribute to diminished muscle hypertrophy and decreased satellite cell proliferation, which is necessary for the muscle regeneration process [16,17,18]. Sainz et al. showed that leptin administration in leptin-deficient mice can trigger muscle cell proliferation by inhibiting myofibrillar protein degradation [19]. However, there is no consistent evidence in human studies that leptin directly increases muscle mass. Furthermore, human obesity is often characterized by leptin resistance accompanied by increased leptin levels, rather than leptin deficiency; this may limit the translation of animal data into clinical interpretation [17,20]. Sarcopenic obesity is an age-related disease defined by the concurrence of an excessive amount of adipose tissue and a decline in muscle mass [21,22,23]. Increased pro-inflammatory cytokines, intramyocellular accumulation of lipids, and macrophage infiltration enhance the muscular atrophy and lipotoxicity mechanisms [22,24,25]. All these cytokines secreted by adipose tissue can enter into the bloodstream and alter the blood cell count, flow rate, and specific biomarker levels [26,27]. The adhesion and transmigration of blood monocytes are triggered by elevated leptin levels [28,29]. Enhanced pro-inflammatory cytokines and leptin levels could also induce leukocytosis [30,31]. A positive correlation exists between BMI and the bloodstream’s leukocyte count [29,32,33]. Considering all this information, obesity is a core disorder in the body that could influence a wide range of tissues or organs. Hwaung and colleagues suggest that obesity is not only defined by the excess amount of adipose tissue but also by increases in these additional “companion” organs and tissues [14].

Many studies investigate the association between obesity and tissues or organs individually. Nevertheless, there are not enough studies regarding the interaction between different organs or tissues and obesity using an integrative approach. Previous studies in the literature have examined the relationship between gene expression in different tissues and obesity, or the interaction between adipose tissue and other tissues. However, no study has yet been found that fully encompasses the gene expression profiles of muscle, blood, liver, visceral adipose, and subcutaneous adipose tissues. With this objective, we tried to identify candidate biological molecules for the treatment or prevention of obesity by examining the gene profiles of different organs and tissues. We aimed to comparatively investigate changes in gene expression associated with obesity using transcriptomic datasets from muscle, blood, liver, visceral adipose, and subcutaneous adipose tissues. DEGs were determined separately for each tissue, and then genes commonly found across tissues were identified. A protein–protein interaction network was constructed from these common genes, and structurally prominent nodes of the network were defined based on degree and betweenness criteria. The study offers an integrative perspective by evaluating multiple tissue transcriptome data together with network-based analysis approaches, and by considering biological processes at the system level rather than the individual gene level. The findings of this study are intended to contribute to a better understanding of molecular mechanisms and to identify candidate targets for further experimental or clinical studies. Furthermore, host–pathogen interaction network analyses performed on the same protein cluster allow for the systematic examination of the intersection points of obesity-related molecular networks with potential pathogenic processes; this approach contributes to the exploratory evaluation of biological risk patterns that may accompany obesity.

## 2. Materials and Methods

### 2.1. Collection of Transcriptome Datasets of Obesity

The transcriptome datasets and raw data were acquired from Gene Expression Omnibus (GEO) [34] and investigated to determine gene expression representation in obesity (Table 1). The specimens in these datasets were collected from subcutaneous and visceral adipose tissue, blood, skeletal muscle, and liver.

### 2.2. Identification of DEGs

Each dataset was analyzed independently under the R/Bioconductor (www.bioconductor.org, accessed on 22 September 2025) software platform to reveal differential gene expression levels in obese patients. During the analysis, RMA normalization [48] and linear models (LIMMA) [49] methods were used for microarray data. Correction for multiple hypothesis testing was performed using the False Discovery Rate (FDR) method, and differentially expressed genes were identified following statistical analysis of each dataset. A *p*-value threshold of ≤0.05 was applied, and a fold change criterion corresponding to a 20% change was used to determine the direction of gene expression alterations.

This method, presented for the detection of genes with different expressions, has been used many times on different diseases by our research group and has given important results [50,51,52,53,54].

### 2.3. Functional Enrichment Analysis

The DEGs list was uploaded to the Metascape [55] web tool, and gene enrichment analyses were performed. GeneSymbol identifiers were used as the basis for uploading gene names to the system. Kyoto Encyclopedia of Genes and Genomes (KEGG) [56], Reactome Gene Sets, WikiPathways (WP) [57], and Gene Ontology (GO) terminology [58] were utilized as annotation sources. Metascape’s default parameters were applied in the analyses. The Benjamini correction was applied as the method for multiple testing adjustment, and enrichment results with *p*-values less than 0.05 were deemed statistically significant. The background gene set was taken to include all human genes defined on the platform. Enriched terms were reported according to their biological significance.

### 2.4. Disease Enrichment Analysis

Disease and gene associations were obtained via Metascape [55] from DisGeNET [59]. DisGeNET is an open-source tool that includes genes and their variant correlations with disorders and incorporates data from diverse curated databases. The Benjamini correction was applied as the method for multiple testing adjustment, and enrichment results with *p*-values less than 0.05 were considered statistically significant.

### 2.5. Protein-Protein Interaction (PPI) Subnetworks

StringApp (version 2.0.1; Swiss Institute of Bioinformatics, Lausanne, Switzerland) performed the physical protein–protein interaction network analysis [60]. StringApp is a plugin created by combining Cytoscape (version 3.10.1; Cytoscape Consortium, San Diego, CA, USA) [61] and STRING [62] bioinformatics tools. It facilitates the transformation of protein–protein interaction data retrieved from STRING into a network using the Cytoscape application. STRING is a web-based open-source database that contains all known and predicted protein–protein interaction (PPI) networks, and it was utilized to investigate the physical and functional interactions between proteins [62]. PPI networks of the DEGs were constructed with a confidence score of ≥0.4 with STRING. After removing unconnected nodes, the DEG PPI networks were visualized using Cytoscape. The top 10 hub proteins in the network were determined with the cytoHubba (version 0.1; National Chiao Tung University, Hsinchu, Taiwan) [63] plugin using the betweenness and degree topological analysis method to identify feature nodes and hub proteins from all protein–protein interaction networks.

### 2.6. Reconstruction of Host-Pathogen Interaction Network

The host–pathogen protein interaction networks were downloaded from the Host–Pathogen Interaction Database (HPID2) [64], which includes experimental and predicted interactions based on literature curation, and the Pathogen–Host Interaction Search Tool (PHISTO) [65], which compiles host–pathogen protein interactions. These databases were explored to identify potential pathogen interactions of candidate proteins associated with obesity.

Protein lists derived from DEGs were matched with human–pathogen interaction records in both databases; duplicate records were removed, and gene symbols were standardized. Analyses were conducted within a hypothesis-generating framework for identifying potential interaction patterns; no quantitative thresholding or statistical background correction based on confidence levels of interactions was applied. Therefore, the resulting network structures represent possible molecular contact points and should not be used to directly infer infection risk, exposure level, or causal relationships.

### 2.7. Reporter Metabolites Associated with T2D

DEGs were uploaded into the Recon3D Human Metabolic Network (version 1.0; Systems Biology Research Group, University of California San Diego, La Jolla, CA, USA) [66] via the Raven Toolbox (version 2.5.0; Chalmers University of Technology, Gothenburg, Sweden) [67] in the MATLAB (version R2021a; MathWorks Inc., Natick, MA, USA) [68] environment, and statistically significant reporter metabolites were explored according to their *p*-values. The Benjamini correction was used for multiple testing adjustments, and reporter metabolites with *p*-values less than 0.05 were considered statistically significant. Common reporter metabolites that are found in all tissues or organs were determined for further analysis.

## 3. Results

### 3.1. Identifying DEGs Through Diverse Tissues

We investigated the 14 different gene expression datasets belonging to liver, blood, skeletal muscle, visceral, and subcutaneous adipose tissue for obesity (Table 1). Each sample was chosen and grouped according to the BMI value of participants. The obese group was defined as those with a BMI greater than 30, while the non-obese group was defined as those with a BMI less than 25. Detailed clinical information for the selected datasets was presented in Supplementary File S1. The results of the statistical analysis of all datasets were presented in Table 2, while DEG lists of each dataset were presented in Supplementary File S2. Common genes across tissues were identified by combining gene lists differentially detected in each tissue using a union approach. Considering that the molecular responses of different tissues to obesity can be heterogeneous in terms of direction and magnitude, it was not required that a gene show the same direction (up- or down-regulated) in all tissues to be considered “common.” Therefore, genes with varying direction across tissues were also included in the analyses to reflect biological heterogeneity. Different platforms, sample sizes, and experimental designs were considered to affect overlap patterns, and this was evaluated within the limitations of the study. Common gene analyses aim to identify recurring differential signals across tissues, rather than directly comparing quantitative effect sizes.

**Figure 1:**
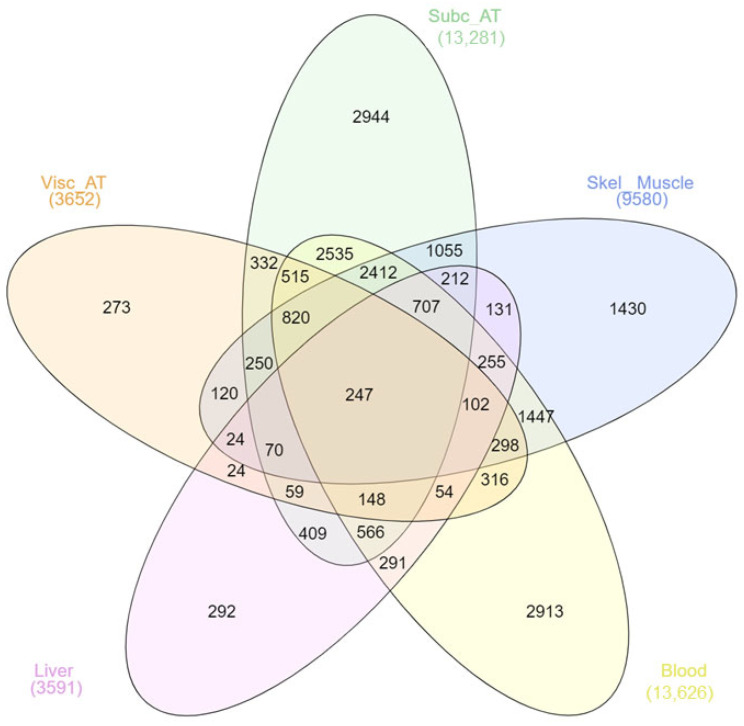
Comparative analysis of DEGs in all tissues/organs (Subc_AT: Subcutaneous Adipose Tissue, Visc_AT: Visceral Adipose Tissue, Skel_Muscle: Skeletal Muscle).

Afterwards, in the comparative analysis of DEGs belonging to different tissues/organs, the skeletal muscle, subcutaneous adipose tissue, and blood stand out due to the number of DEGs they possess, although liver and visceral adipose tissue have lower amounts. The DEG results also demonstrated that the most affected tissues/organs are the subcutaneous adipose tissue and blood due to their high numbers of DEGs. Despite the large quantity of DEGs in obesity data collections, only 247 genes are common in all tissues and organs (Figure 1).

**Figure 2:**
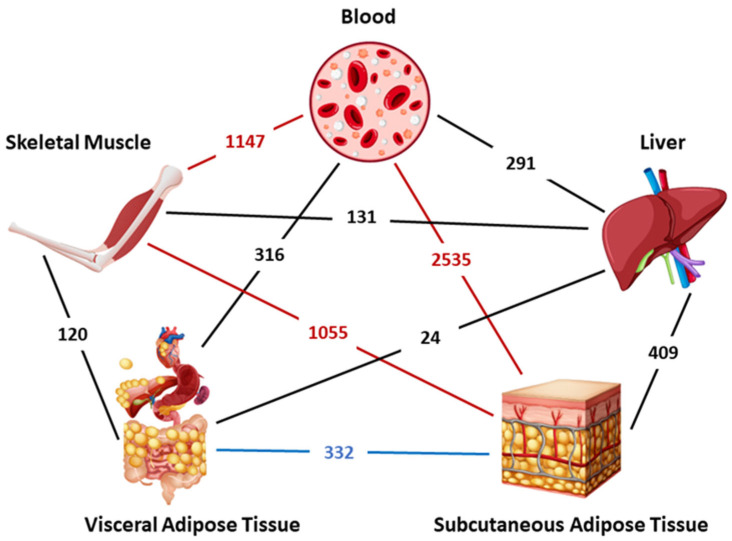
Common DEGs between only two different tissues/organs.

Cumulative analysis indicated that there were also shared genes over several tissues/organs. The triangle skeletal muscle, subcutaneous adipose tissue, and blood had the highest number of interacting DEGs, and interestingly, visceral and subcutaneous adipose tissue were limited. It is noteworthy that the liver, which is closely related to adipose tissue, shares only a few common genes with both visceral and subcutaneous adipose tissue. Even though subcutaneous adipose tissue and muscle tissue have a higher number of common DEGs, there were a modest number of DEGs for visceral adipose tissue and muscle tissue. Blood and subcutaneous adipose tissue interaction comes to the forefront with the highest number of DEGs (Figure 2).

**Figure 3:**
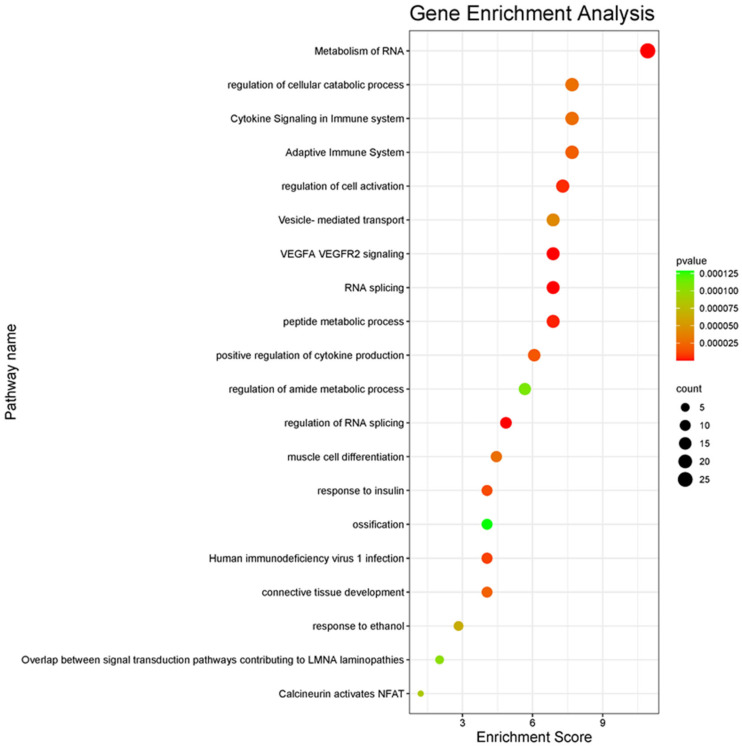
Gene set enrichment analysis of common DEGs.

To clarify the key mechanisms and pathways, we performed the gene set enrichment analysis for these 247 common genes, and the top 20 related pathways were presented in the heatmap (Figure 3). While the most related pathway is metabolism of RNA, immune system and cytokine production-related pathways emerge prominently. Response to insulin and muscle cell differentiation are the other remarkable pathways in which DEGs are mainly involved. COL6A1, Glutamic-Pyruvate Transaminase Alanine Aminotransferase (GPT), Leptin (LEP), Pyruvate Dehydrogenase Kinase 4 (PDK4), SGCB, SREBF, STXBP3, SORBS, RHOQ, and Transient Receptor Potential Cation Channel Subfamily V Member 4 (TRPV4) belong to the response to the insulin pathway. The *LEP* gene encodes the leptin protein, which is secreted by adipose tissue and regulates energy homeostasis. It acts as a satiety controller and is directly related to obesity [69]. It also plays a role in insulin signaling, inhibits insulin secretion, and improves insulin sensitivity [70]. *GPT* has an important effect on glucose and amino acid metabolism and encodes the cytosolic alanine aminotransaminase 1 (ALT1) enzyme. The serum level of ALT is used as a clinical marker for liver function [71,72]. The *PDK4* gene encodes a mitochondrial protein, mainly expressed in the heart, skeletal muscle, liver, and kidney. It plays a crucial role in glucose and lipid metabolism [73,74]. It is reported that this gene might be a therapeutic candidate for muscular atrophy [74]. *TRPV4* belongs to the transient receptor potential channels superfamily and is expressed in pancreatic beta cells [75,76]. It triggers insulin secretion and has a key role in beta cell production in the body [76,77]. *LEP* and *TRPV4* are also present in the positive regulation of the cytokine production pathway. *TRPV4* is engaged in inflammation processes [78], and it is reported that the anti-TRPV4 treatment reduces inflammation and may be a therapeutic candidate for some diseases that cause inflammation [78,79]. Leptin is known as an inflammatory regulator in obesity that may cause multicausal diseases [80]. Leptin has a comparable structure with some cytokines such as IL-6, IL-12, etc. In the case of increased levels of leptin like hyperleptinemia is associated with increased proinflammatory cytokine profiles [81,82].

### 3.2. Gene–Disease Association Analysis

**Figure 4:**
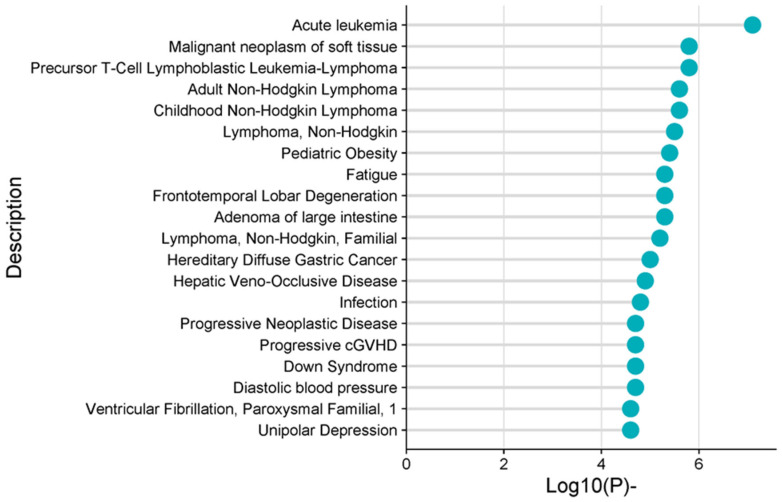
Disease enrichment analysis.

As a result of the tissue-based examination of obesity, disease associations revealed that obesity is most significantly associated with cancer and that this association is accompanied by leukemia, lymphoma, and gastric cancer. Acute leukemia is the most strongly correlated with obesity, and lymphoma is ranked second. We also noticed that there are several subtypes of lymphoma: adult and childhood non-Hodgkin lymphoma and T-cell lymphoblastic leukemia–lymphoma. Infection is another significantly correlated disease according to the gene–disease association analysis (Figure 4). Similar to this result, we also found that various immune system-related pathways stand out in gene set enrichment analysis. Another important trait is that diastolic blood pressure is correlated with obesity.

### 3.3. Protein–Protein Interaction (PPI) Networks

**Figure 5:**
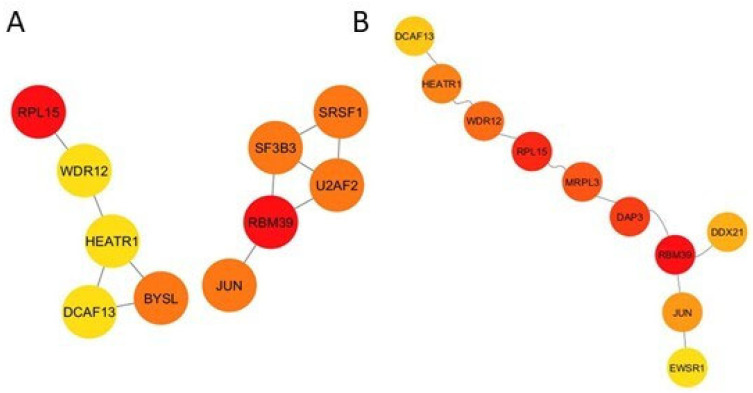
Top 10 hub genes according to the degree topological metric (**A**) and the betweenness topological metric (**B**).

Following the DEG analysis, we uploaded all tissue-shared DEGs to the StringApp in Cytoscape and selected only the physical subnetwork. The protein–protein interaction subnetwork had 246 nodes and 113 edges. The top 10 hub proteins were obtained by performing topological analysis for this subnetwork. We used degree as a local-based metric and betweenness centrality as a global-based metric, both local and global attributes to identify the hubs, and both local and global features of the nodes within the graphs were examined simultaneously. Ribosomal Protein L15 (RPL15) and RNA Binding Motif Protein 39 (RBM39) are the top proteins in both degree and betweenness metrics. Jun Proto-Oncogene (JUN), DDB1 and CUL4 Associated Factor 13 (DCAF13), WD Repeat Domain 12 (WDR12), and HEAT Repeat Containing 1 (HEATR1) are other hub proteins in the network (Figure 5A,B).

### 3.4. Host–Pathogen Interaction Network

**Figure 6:**
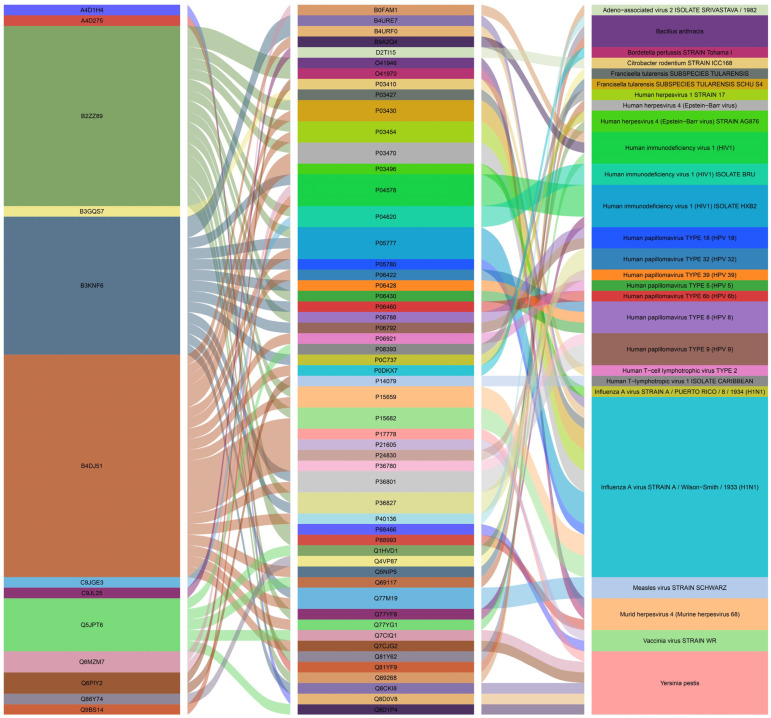
Host–pathogen interaction network.

After obtaining proteins belonging to all tissue-shared DEGs, we retrieved the whole host–pathogen protein interaction data and determined the pathogen proteins and organisms that interacted with our protein list. The entire network included 100 different host–pathogen protein interactions. These interactions comprised 13 unique host proteins, 54 unique pathogen proteins, and 27 unique pathogen organisms. *Influenza A virus* had the highest interaction with 18 different host–pathogen protein interactions. Subsequently, *Bacillus anthracis* and *Yersinia pestis* were ranked second and third in the network, respectively, with 13 and nine different host–pathogen protein interactions. Eleven unique proteins belonging to the *Influenza A virus* interacted with four unique human proteins (Figure 6).

### 3.5. Metabolic Fingerprints of Different Tissues Associated with Obesity

Reporter metabolites analysis revealed that there were only a small number of common metabolites in all tissues. 2-Oxoglutarate and Adenosine Triphosphate are respiratory system products produced in the Krebs cycle by leaving CO_2_ from isocitrate. Succinate and D-mannose are other metabolites shared with all tissues. Succinate is also a Krebs cycle byproduct, and D-mannose is a natural monosaccharide. It is known that D-mannose is an epimer of D-glucose. Following all tissue-shared metabolites, it is observed that D-glucose is found among the metabolites present in at least four tissues/organs except visceral tissue. Cholesterol and sphingosine were also present in at least four of the cases, except for the muscle. Keto-phenylpyruvate, protoheme, and acetone are liver, muscle, and blood-associated metabolites. Acetone is a kind of ketone. Keto-phenylpyruvate and protoheme are *Saccharomyces cerevisiae*-associated metabolites.

## 4. Discussion

Common and tissue-specific DEGs, proteins, and reporter biomolecules were investigated across different tissues in obesity via analysis of transcriptomics datasets with human biological networks to elucidate the key mechanisms underlying obesity. It is notable that despite divergence, the genetic profiles of tissues and organs exhibit commonalities. Particularly, analysis of statistically significant changes in gene expression reveals that subcutaneous adipose tissue and blood are the areas most significantly affected by obesity. In their 2019 study, Ronquillo and colleagues reported that subcutaneous and visceral adipose tissues possess markedly distinct genetic profiles [83].

The analysis of transcriptome datasets related to obesity identified 247 genes that are commonly expressed across all tissues and organs. Gene enrichment analysis to determine the pathways these genes are involved in revealed that RNA metabolism pathways are the most prominent. Additionally, Human Immunodeficiency Virus 1 (HIV-1) infection is another notable pathway that is firmly associated with the DEG list. This outcome indicates that the tissues and organs in the body respond to the HIV infection and elicit an immune response, possibly modifying the sensitivity of the body to the viruses within the framework of obesity. There is a significant connection between HIV infection and obesity, and there are many studies about it in the literature [84,85,86]. A study in 2023 investigated HIV patients from 2014 to 2020. It is indicated that the prevalence of overweight or obese individuals among these people living with HIV (PLWH) increased consistently [86]. Another study conducted by Tate et al. demonstrated that in the South, a significant proportion of HIV patients are either overweight or obese [84,87]. Additionally, the Adaptive Immune System and Cytokine Signaling in the Immune System pathways have important functions in creating a response to pathogens in the body, and we found that they are significantly associated with obesity. Functional enrichment analysis of these shared DEGs in this study raises the possibility of a bidirectional relationship between obesity and infectious processes, whereby obesity-induced immune dysregulation may increase susceptibility to infections, while prior or chronic pathogen exposure may contribute to obesity-associated inflammation. To explore this hypothesis, we constructed a host–pathogen interaction network to systematically examine the molecular interfaces between obesity-associated host genes and pathogen-related factors.

Consistent with our findings, which show common DEGs enriched in adaptive immune responses, cytokine signaling, and viral infection-related pathways across obesity-associated tissues, there is growing evidence supporting a bidirectional relationship between obesity and infectious processes. Epidemiological studies indicate that obesity is associated with immune dysregulation, chronic low-grade inflammation, and altered adipocytokine signaling, which collectively increase susceptibility to infections and influence disease severity, while normal body weight is associated with the lowest infection risk [87]. Mechanistically, metabolic complications and leptin-driven immune imbalance in obesity have been shown to impair antiviral responses and vaccine effectiveness, further influencing obese individuals to viral infections [88]. Conversely, the concept of infectobesity proposes that certain pathogens may directly contribute to obesity development, with experimental and clinical evidence implicating viruses such as human adenovirus-36 in adiposity through inflammatory and metabolic reprogramming [89]. In the last two decades, researchers have studied the concept of infectobesity. For example, recent population-based data further demonstrate that HAdV-36 seropositivity is associated with increased systemic inflammation, oxidative stress–antioxidant imbalance, and lipid alterations independent of body mass index, suggesting virus-driven immunometabolic effects that may promote white adipose tissue accumulation [90]. In our study, we have also showed that Adeno associated virus 2, HIV, Human Papillomavirus (HPV), and more viral and bacterial sources may contribute to obesity through the host–pathogen PPIs.

Taken together, these findings, supported by our host–pathogen interaction network analysis, highlight a reciprocal model in which obesity-related immune dysfunction enhances vulnerability to infections, while pathogen exposure may, in turn, contribute to obesity-associated molecular remodeling.

All these outcomes indicate that obesity may influence the mechanisms protecting against the foreign molecules or microbes in all tissues and organs that were analyzed in this study, consequently affecting the immune system of the body overall.

The gene set enrichment analysis stated that significant variations were observed in the insulin response-associated mechanisms of obesity in tissues/organs. It is well-determined that an excessive amount of adipose tissue in the body causes the development of insulin resistance over time [91,92]. The release of glucose from the liver is disrupted during obesity. This disruption causes the tissues and organs such as adipose and skeletal muscle to be unable to uptake the glucose inside the cells. Therefore, insulin resistance develops in the body [93,94]. Additionally, free fatty acid (FFA) can also trigger insulin secretion from the pancreas, and the elevated level of FFA caused by increased lipolysis from adipose tissue may affect insulin resistance during obesity [94,95,96,97].

In line with our results, it is reported that cytokine production is enhanced by the excessive amount of adipose tissue during obesity, especially pro-inflammatory ones [98,99]. Adipose tissue can crosstalk with other tissues and organs in the body and can influence the functions directly [100,101,102,103].

The topological analyses of the protein–protein interaction network revealed the top 10 hub proteins across all tissues/organs. Ribosomal protein L15 (RPL15) is marked with 1 in both degree and betweenness analysis. It is a component of the 60S subunit of the ribosome and has key roles in pre-rRNA processing and ribosomal assembly [104,105]. It is reported that the RPL15 is significantly upregulated in gastric cancer tissues and cell lines [106]. RPL15 is also associated with different types of brain disorders [107,108]. Kim et al. 2016 found that there was a notable association between Parkinson’s disease (PD) and *RPL15* expression; it was downregulated in the substantia nigra of PD patients [107]. Another study conducted in 2016 found that *RPL15* is distinctively expressed in regions of the brain in Alzheimer’s Disease [108]. Nevertheless, there are no studies on the obesity relation with *RPL15* expression. With this study, we reported for the first time that there was a marked association between the *RPL15* gene and obesity. We believe that this gene, which is expressed at a common level in all tissues and organs, has an important place in elucidating the key mechanisms of obesity’s interaction between tissues. RNA-binding motif protein 39 (RBM39) is another hub protein that was associated with some types of cancer-modulating transcription of many tumor-related genes [109,110,111,112]. There was only one study that showed the interaction between RBM39 and maternal obesity in sheep [113]. We reported that *RBM39* is a key gene for organ crosstalk during obesity in humans for the first time. Human HEAT repeat containing 1 protein (*HEATR1*) [114,115], Bystin-like (*BYSL*) [116,117], WD-repeat protein 12 (*WDR12*) [118], and *DDB1* and *CUL4* associated factor 13 (*DCAF13*) [119,120] genes also show similar association with various cancer types, and their relationship with obesity was revealed for the first time in this study. Similar to previous studies in the literature, we found that Serine-arginine splicing factor 1 (*SRSF1*) [121], Splicing Factor 3b Subunit 3 (*SF3B3*) [122], and U2 small nuclear RNA auxiliary factor 2 (*U2AF2*) [123] are among the important genes associated with obesity.

Host–pathogen analysis, which was performed in this study, was not intended to make direct inferences about susceptibility or exposure levels to specific pathogens, but rather to generate exploratory clues about where obesity-related molecular networks might intersect with host–pathogen interactions reported in the literature. When we examine the results of the host–pathogen interaction analysis, it is seen that viral pathogens are the most common in the pathogen list and are accompanied by bacterial species. In the gene enrichment analysis, the immune system-related pathways are especially highlighted, and these results suggest that obesity may trigger important problems in the body’s protection mechanism for all these organs and tissues. It is reported that there is a strong interaction between the influenza A virus, HIV, and obesity [88,89]. *Bacillus anthracis* is a bacterial pathogen that stands out on our list, and although it occurs mostly in farm animals and wild animals, it is seen in humans on rare occasions and is known to cause anthrax disease [124,125]. For the first time, we declared the relationship between obesity and *Bacillus anthracis* infection in this study. *Yersinia pestis* is another important pathogen candidate and is also known as a zoonotic pathogen. It is reported that it causes bubonic plague and can be transmitted to humans through flea bites [126]. It has also been shown that consumption of undercooked goat and camel meat can cause oropharyngeal plague [127]. Its relationship with obesity has not been studied before, but it was revealed for the first time in this study. Considering all this information, it seems likely that people will be exposed to these infections as their diet shifts towards more animal-based foods, especially meat, along with obesity.

Succinate is a metabolite that has regulatory roles in obesity, but it is not yet known whether it has a beneficial effect on the obesity progression of obesity. A study conducted in 2023 showed that succinate supplementation could reduce both visceral and subcutaneous cell size and trigger brown adipose tissue activity. However, Carolina et al. indicated that obesity is associated with increased succinate levels [128,129]. D-mannose is another common metabolite in all tissues/organs. It has anti-inflammatory effects on the body and has many beneficial effects on inflammatory diseases [130]. D-mannose can also trigger fatty acid oxidation and prevent diet-induced obesity [131].

This study is exploratory in nature and requires the interpretation of findings at the correlation level. In particular, the fact that the publicly available datasets used in these analyses were generated on different platforms, and that their sample sizes and experimental designs differed, inevitably led to limitations such as data heterogeneity and potential batch effects. Furthermore, since individual clinical variables such as age, gender, comorbidities, and medication use were not consistently reported across many datasets, detailed comparisons across clinical subgroups were not possible.

The analyses conducted within the scope of this study, using network-based networks and host–pathogen interaction data, revealed prominent gene candidates and inferences that allow for the system-level consideration of complex molecular patterns associated with obesity. In this respect, the study goes beyond analyses based on individual datasets, offering an integrative perspective across different biological layers and generating new comparative observations on interaction networks that have been addressed only to a limited extent in the literature. From a clinical perspective, while the findings are not intended to directly translate into diagnostic, prognostic, or treatment decisions, the proposed candidate network structures and gene clusters provide a rational starting point for further mechanistic studies and experimental validation research. Future studies are expected to further enhance the biological and translational value of these findings through validation analyses in independent and well-defined clinical cohorts, molecular investigations supported by functional experiments, and the integration of additional layers such as proteomics or metabolomics.

## 5. Conclusions

Although it is known that there is a relationship between obesity and ribosomal biogenesis, and that ribosomal biogenesis is triggered to meet increased demand in obese individuals, a specific relationship between these two genes and obesity has not been previously identified. This study reports a possible association between the RPL15 and RBM39 genes and obesity for the first time. The common prominence of these genes in various tissues and organs, such as muscle, blood, liver, visceral adipose, and subcutaneous adipose tissues, reinforces the importance of these candidates for obesity at a systemic level. Further studies detailing the relationship between these genes and obesity are believed to be promising for elucidating the mechanism of the relationship between obesity and ribosomal biogenesis, and even confirming this relationship holds promise for the treatment or prevention of obesity.

Based on the analysis, it is thought that obesity directly affects the immune system-related molecular mechanisms that the body uses to defend against foreign molecules or microbes, and that the body becomes more vulnerable to external harmful molecules. It is seen that with the changing cytokine profile associated with obesity, the body cannot create a correct response to circulating glucose or use it correctly within the cell, and disruptions in molecular mechanisms related to insulin may occur in the body. According to the host–pathogen interaction analysis, it was determined that viral infections may occur more frequently with obesity. In addition, this study reports for the first time that the body may become more susceptible to infections caused by *Bacillus anthracis* and *Yersinia pestis*. As a result of our study, it is thought that the effects of Succinate, which is related to obesity, on obesity should be determined in more detail, and it is thought that clarifying this relationship might be important for the treatment or prevention of obesity.

This study employs an exploratory and correlational analytical framework, using multiple data integration and network-based approaches to systematically elucidate potential relationships between genes, biological processes, and interactions; it does not aim to generate clinical predictions for disease treatment. The findings should be considered a starting point for hypotheses and candidate molecular targets requiring further experimental validation.

## Figures and Tables

**Table 1:** Transcriptomics Datasets.

GEO ID	Tissue/Organ Name	References
GSE48964	Subcutaneous Adipose Tissue	[35]
GSE25401		[36]
GSE27951		[37]
GSE24883		[38]
GSE59034		[39]
GSE24883	Visceral Adipose Tissue	[38]
GSE88837		[40]
GSE45745	Skeletal Muscle	[41]
GSE22435		[42]
GSE474		[43]
GSE109597	Blood	[44]
GSE18897		[45]
GSE48452	Liver	[46]
GSE15653		[47]

**Table 2:** Transcriptomics Datasets and Statistical Analysis Results for DEGs.

GEO ID	Tissue/ Organ Name	# of Down-Regulated DEGs	# of Up- Regulated DEGs	# of Total Regulated DEGs	# of Sample Ob/Nonob	References
GSE48964	Subcutaneous Adipose Tissue	953	784	2482	3/3	[35]
GSE25401		409	806	5082	30/26	[36]
GSE27951		1173	1126	2419	6/3	[37]
GSE24883		1468	291	1759	8/8	[38]
GSE59034		1286	2106	8828	16/16	[39]
GSE24883	Visceral Adipose Tissue	623	151	774	8/8	[38]
GSE88837		1397	1164	3001	15/14	[40]
GSE45745	Skeletal Muscle	3014	3464	6691	5/6	[41]
GSE22435		1924	1482	3470	3/3	[42]
GSE474		84	48	1319	16/8	[43]
GSE109597	Whole Blood	955	121	6177	20/43	[44]
GSE18897		3010	3868	10,537	20/20	[45]
GSE48452	Liver	864	787	2302	16/16	[46]
GSE15653		748	731	1479	4/3	[47]

## Data Availability

The datasets that were used in this study are publicly available at Gene Expression Omnibus (GEO Database) with the following links: GSE48964—https://www.ncbi.nlm.nih.gov/geo/query/acc.cgi?acc=GSE48964 (accessed on 17 March 2024); GSE25401—https://www.ncbi.nlm.nih.gov/geo/query/acc.cgi?acc=GSE25401 (accessed on 14 March 2024); GSE27951—https://www.ncbi.nlm.nih.gov/geo/query/acc.cgi?acc=GSE27951 (accessed on 17 March 2024); GSE24883—https://www.ncbi.nlm.nih.gov/geo/query/acc.cgi?acc=GSE24883 (accessed on 17 March 2024); GSE59034—https://www.ncbi.nlm.nih.gov/geo/query/acc.cgi?acc=GSE59034 (accessed on 17 March 2024); GSE88837—https://www.ncbi.nlm.nih.gov/geo/query/acc.cgi?acc=GSE88837 (accessed on 17 March 2024); GSE45745—https://www.ncbi.nlm.nih.gov/geo/query/acc.cgi?acc=GSE45745 (accessed on 17 March 2024); GSE22435—https://www.ncbi.nlm.nih.gov/geo/query/acc.cgi?acc=GSE22435 (accessed on 20 March 2024); GSE474—https://www.ncbi.nlm.nih.gov/geo/query/acc.cgi?acc=GSE474 (accessed on 20 March 2024); GSE109597—https://www.ncbi.nlm.nih.gov/geo/query/acc.cgi?acc=GSE109597 (accessed on 20 March 2024); GSE18897—https://www.ncbi.nlm.nih.gov/geo/query/acc.cgi?acc=GSE18897 (accessed on 17 March 2024); GSE48452—https://www.ncbi.nlm.nih.gov/geo/query/acc.cgi?acc=GSE48452 (accessed on 17 April 2024); GSE15653—https://www.ncbi.nlm.nih.gov/geo/query/acc.cgi?acc=GSE15653 (accessed on 18 April 2024).

## Supplementary Materials

The following supporting information can be downloaded at: https://www.mdpi.com/article/10.3390/metabo16020095/s1; Supplementary File S1: Metadata of transcriptome datasets. Supplementary File S2: The DEGs lists of transcriptome datasets.
